# Safety, tolerability, and pharmacokinetics/pharmacodynamics of JMT103 in patients with bone metastases from solid tumors

**DOI:** 10.3389/fonc.2022.971594

**Published:** 2022-08-05

**Authors:** Xu Liang, Junli Xue, Xiaoxiao Ge, Jin Li, Huiping Li, Liqiong Xue, Lijun Di, Wenbo Tang, Guohong Song, Qun Li, Hanfang Jiang, Wei Zhao, Fengjuan Lin, Bin Shao, Xiugao Yang, Zhufeng Wu, Tianyi Zhang, Chenchen Wang, Ye Guo

**Affiliations:** ^1^ Key Laboratory of Carcinogenesis and Translational Research (Ministry of Education/Beijing), Department of Breast Oncology, Peking University Cancer Hospital and Institute, Beijing, China; ^2^ Department of Oncology, Shanghai East Hospital, School of Medicine, Tongji University, Shanghai, China; ^3^ Clinical Sciences Division, CSPC ZhongQi Pharmaceutical Technology (Shijiazhuang) Co., Ltd, Shijiazhuang, China

**Keywords:** bone metastasis, JMT103, RANKL, phase 1 study, pharmacokinetics, N-telopeptide

## Abstract

Bone metastases are common complications of solid tumors. The outcome is poor despite major progress in cancer therapies. We describe a multicenter, open-label, phase 1, dose escalation and expansion trial of JMT103, a novel fully humanized receptor activator of nuclear factor kappa-B ligand (RANKL)-targeting monoclonal antibody, in adults with bone metastases from solid tumors. The study assessed the safety, tolerability, and pharmacokinetics/pharmacodynamics of JMT103. Patients received JMT103 at doses of 0.5, 1.0, 2.0, and 3.0 mg/kg every 4 weeks for 3 cycles. Among 59 patients enrolled, 20 and 39 patients participated in the dose-escalation and dose-expansion phases, respectively. One dose-limiting toxicity was observed at 2.0 mg/kg. The maximum tolerated dose was not determined. Treatment-related adverse events were reported in 29 (49.2%) patients, most commonly hypophosphatemia (30.5%), hypocalcemia (23.7%), and hypermagnesemia (10.2%). No treatment-related serious adverse events were reported. Two patients died due to disease progression, which were attributed to gastric cancer and lung neoplasm malignant respectively. Dose proportionality occurred between exposure levels and administered dose was within a dose range of 0.5 to 3.0 mg/kg. The suppression of urinary N-telopeptide corrected for creatinine was rapid, significant, and sustained across all doses of JMT103, with the median change from baseline ranging from –61.4% to –92.2% at day 141. JMT103 was well tolerated in patients with bone metastases from solid tumors, with a manageable safety profile. Bone antiresorptive activity shows the potential of JMT103 for treatment of bone metastases from solid tumors.

Registration No.: NCT03550508; URL: https://www.clinicaltrials.gov/

## Introduction

Bone metastases are frequent complications of solid tumors and occur in more than 1.5 million patients worldwide, of which 65–80% have metastases from breast or prostate cancers (1–4). The incidence of bone metastases is also increasing in other cancers, such as multiple myeloma, hepatocellular carcinoma, lung cancer, and malignant melanoma (5–7). Bone metastases often result in devastating skeletal-related events (SREs), including bone pain, pathologic fractures, spinal cord compression, bone radiotherapy, orthopedic surgery, and hypercalcemia, contributing substantially to both morbidity and mortality in patients with advanced cancer (3, 5, 8, 9). Despite major progress in cancer therapies, the median survival from diagnosis of bone metastasis is within the range of 6–53 months, and the 5-year survival in breast cancer remains around 20% (10, 11).

Therapy for bone metastasis includes external beam radiotherapy, systemic therapy with cytotoxic antineoplastic medications (chemotherapy) and endocrine agents, targeted therapies, targeted radionucleotide therapies, and recent novel palliative treatments, such as high-intensity focused ultrasound and radiofrequency thermal ablation, that are beneficial in relieving the pain associated with bone metastases and improving the quality of life (4, 12–14). Bone-targeted agents such as bisphosphonates and denosumab have become the standard of care for the treatment and prevention of SREs associated with bone metastases from solid tumors (15, 16). However, use of bisphosphonates has the potential risk of adverse effects such as osteonecrosis of the jaw (ONJ), esophageal irritation, and fractures (17); bisphosphonates should be used cautiously in patients with renal insufficiency (17, 18) and the reversible effect after discontinuation of denosumab (19) should be considered. Therefore, it is important to develop new therapies to improve patients’ outcomes.

JMT103 (JMT-BIO, Shanghai, China) is an innovative, fully humanized monoclonal antibody that was developed by replacing the Fc end of denosumab from immunoglobulin (Ig) G2 to IgG4, thus significantly improving uniformity and stability (20). JMT103 particularly targets the receptor activator of nuclear factor kappa-B ligand (RANKL) with higher affinity and specificity than that of denosumab (20), thereby inhibiting osteoclastogenesis and osteoclast-mediated bone resorption. As suggested by the preclinical pharmacodynamic studies (JMT-Bio Inc., unpublished data, 2020), JMT103 (at the doses of 2.5 mg/kg and 25 mg/kg) can significantly reduce bone resorption biomarkers of type I collagen carboxyl terminal peptide (β-CTx) and tartrate-resistant acid phosphatase 5b (TRAP5b) in aged female rhesus monkey models of osteoporosis, increase bone mineral density (BMD) in ovariectomized rhesus monkey models of osteoporosis, and significantly reduce serum calcium concentration in both of the models. Moreover, the results of repeated-dose toxicology suggested that JMT103 was safe and tolerable up to the dose of 10 mg/kg after once-weekly subcutaneous (s.c.) injection for 13 weeks. Thus, this study was conducted to evaluate the safety, tolerability, pharmacokinetic (PK) and pharmacodynamic (PD) profiles of JMT103 in patients with solid tumor and bone metastasis.

## Materials and methods

### Patients

Eligible patients were 18–75 years of age with histologically or cytologically confirmed malignant solid tumors and radiographic evidence of at least one bone metastasis. Patients had an Eastern Cooperative Oncology Group performance status of 0 or 1, and life expectancy of at least 7.5 months, as well as adequate renal, liver, and bone marrow function.

Patients were excluded if they reported prior or current osteomyelitis or ONJ; unhealed dental or oral surgery wounds, or acute tooth or jaw disease requiring oral surgery, or therapeutic radiotherapy, orthopedic surgery, or invasive dental surgery planned to be performed during the study; known active brain or leptomeningeal metastases; bone metabolic diseases, rheumatoid arthritis, or hyperparathyroidism or parathyroid dysfunction. Other key exclusion criteria included prior treatment with denosumab or its congeners, or treatment with bisphosphonates within 6 weeks before enrollment; or prior administration of osteoprotegerin or calcitonin, parathyroid hormone-related peptides, mithramycin, or strontium ranelate within 6 months before enrollment; or unresolved toxicities > grade 1 from previous treatment regimens. Patients were also excluded if they reported or had evidence of disorders that could affect bone metabolism; or uncontrolled systemic diseases.

The study protocol was approved by independent ethics committees at each site, and all patients provided written informed consent. The study was performed in accordance with the Declaration of Helsinki and Good Clinical Practice guidelines.

### Study design

This was a multicenter, open-label, phase I study. The study included two phases: a dose-escalation phase and a dose-expansion phase. The primary endpoints were safety and tolerability. The secondary endpoints included PK profile, bone turnover biomarkers, and immunogenicity. The exploratory endpoints were BMD and SREs (not recorded in this study).

In the dose-escalation phase, the following doses were proposed: 0.5, 1.0, 2.0, and 3.0 mg/kg. Across these cohorts, sequential patient groups were administered a single dose of JMT103 to determine the maximum-tolerated dose (MTD). After a washout period of 12 weeks, patients were administered three additional doses of JMT103 every 4 weeks for 3 cycles. Dose escalation was carried out according to an accelerated titration design for doses of 0.5 and 1.0 mg/kg, and *via* the traditional “3+3” design for doses of 2.0 and 3.0 mg/kg. One patient was enrolled in each accelerated titration dose level. The accelerated titration design for dose-escalation was changed to the traditional “3+3” design when dose-limiting toxicities (DLTs) or ≥ grade 2 treatment-related adverse events (TRAEs) were observed. Dose escalation was terminated if the DLT occurred in ≥33% of patients or even if the MTD was not reached at the 3.0 mg/kg dose level.

The highest dose level at which no more than one of six patients had a DLT in the first 4 weeks (the DLT evaluation period) was determined to be the MTD. A DLT was defined as one or more of the following TRAEs or possibly/probably TRAEs occurring in the DLT evaluation period: grade 4 neutropenia lasting >3 days or recurring after symptomatic treatment; febrile neutropenia (absolute neutrophil count <1,000/mm^3^, body temperature >38.3°C, or ≥38°C for at least 1 hour); grade 3 neutropenia with evidential infection; grade 3 thrombocytopenia with bleeding tendency, or ≥ grade 4 thrombocytopenia; ≥ grade 3 hypocalcemia or hypophosphatemia (except for grade 3 hypophosphatemia that improved within 1 week after oral supplements or for which there were no clinical symptoms); ≥ grade 2 ONJ; ≥ grade 3 injection site reaction; ≥ grade 3 allergic reaction; and any other ≥ grade 3 non-hematological toxicities requiring medical intervention, or resulting in hospitalization or prolongation of the hospital stay, or abnormalities lasting for more than 1 week; or adverse events (AEs) that the investigator or sponsor considered inappropriate for further escalation.

JMT103 dose levels,1.0 mg/kg, 2.0 mg/kg, and 3.0 mg/kg were selected in the dose-expansion phase to further explore the durable safety, tolerability, PK, and PD of repeated doses of JMT103. The dose-expansion phase was started at the same dose or lower dose after completion of the tolerance evaluation of one dose level in the dose-escalation phase. Patients were enrolled into one of three cohorts and received three cycles of JMT103.

### Study treatments

JMT103 was administered *via* s.c. injection to the upper thigh, upper arm, or abdomen. Patients in dose-escalation phase received JMT103 at 0.5, 1.0, 2.0, or 3.0 mg/kg on day 1, day 85, day 113, and day 141. Patients in the dose-expansion phase received JMT103 at 1.0, 2.0, or 3.0 mg/kg on day 1, day 29, and day 57. Radiotherapy for bone metastatic lesions of tumors, surgery to bone, and medications affecting bone metabolism were not allowed throughout the study. Antineoplastic therapy (e.g., chemotherapy, hormone therapy, radiotherapy for non-bone metastatic tumors) was allowed during the study period, but the dose adjustment was not allowed within 2 weeks before and after initial JMT103 administration. Chemotherapy was not allowed within 2 weeks before and 1 week after the first dose of JMT103. In a subsequent study period, the interval should be no less than 3 days between the use of JMT103 and chemotherapy. Supportive treatment was also allowed according to routine clinical practice. A daily supplement of ≥400 IU vitamin D was prescribed if the serum 25-(OH)-Vitamin D concentration was <20 ng/kg.

### Safety, PK and PD assessments

Safety assessments were based on all grades of AEs, including serious AEs (SAEs), all deaths, changes in laboratory values, physical findings, vital signs, 12-lead electrocardiogram (ECG), oral examination, and positivity for anti-drug antibodies (ADAs). AEs were assessed using the National Cancer Institute Common Terminology Criteria for Adverse Events (version 4.03). ADA blood samples were collected on days 1, 29, 57, and 141 in the dose-escalation phase and days 1, 29, 57, 141 and 225 in the dose-expansion phase, and were measured by electrochemiluminescence (ECL) using the bridge assay format.

Blood samples for JMT103 PK in the dose-escalation phase were collected as follows: pre-dose on days 1, 85, 113, and 141; post-dose on days 1, 2, 8, 15, 22, 29, 43, 57, 99, 127, 155, 169, 197, and 225. During the dose-expansion phase, for three treatment days, blood samples for JMT103 PK were collected within 4 hours pre-dose and within 4 hours post-dose on days 1 and 57, and pre-dose on day 29; other post-dose blood samples were collected on days 2, 5, 8, 15, 22, 43, 58, 61, 64, 71, 78, 85, 99, 113, and 141. The serum concentrations of JMT103 were measured using enzyme-linked immunosorbent assay (ELISA) method with a lower limit of quantification of 20.0 ng/mL.

For PD analysis, blood samples were collected at each visit in the dose-escalation phase and on days 1, 2, 5, 8, 15, 22, 29, 43, 57, 71, 85, 113, and 141 in the dose-expansion phase. The detection methods of serum biomarkers’ concentrations were as follows: urinary N-telopeptide (uNTx) and TRAP5b were measured by ELISA; serum C-telopeptide I (sCTx-I), bone alkaline phosphatase (bALP) and intact parathyroid hormone (iPTH) were measured by ECL; urinary creatinine (Cr) was measured by the enzymic method; serum albumin was measured by the bromocresol green method; serum calcium was measured by colorimetry and adjusted by serum albumin.

### Statistical analyses

We estimated that up to 6 and 15 patients would be enrolled in each dose cohort in the two phases. Sample size was estimated without statistical hypothesis. The safety and tolerability were assessed in the safety analysis set (SAS), which was defined as all patients who received at least one dose of JMT103. The DLT analysis set (DLTAS), which was used to calculate the DLT proportion in each cohort, included all SAS patients who received at least 80% of the study drug and completed evaluation during the 4 weeks after first dosing in the dose-escalation phase. The PK profile was characterized in the PK analysis set (PKAS), which included patients with at least one PK variable. Biomarkers of bone metabolism were characterized in the PD analysis set (PDAS), which included patients with evaluable PD results.

Descriptive statistics were used to summarize the safety, demographics, and baseline characteristics. PK parameters were calculated based on actual sampling timepoint records and were estimated using noncompartmental analysis in Phoenix WinNonlin (version 7.0; Pharsight Corporation, Cary, NC, USA). From the serum concentration-time curve, the PK parameters after the first and multiple administrations were determined. A power model was used to explore the dose proportionality of JMT103 if there were effective concentration data in ≥3 dose cohorts. All the statistical analyses were performed using the SAS software (version 9.4, SAS Institute, NC, USA).

## Results

### Patient demographics

**Figure 1:**
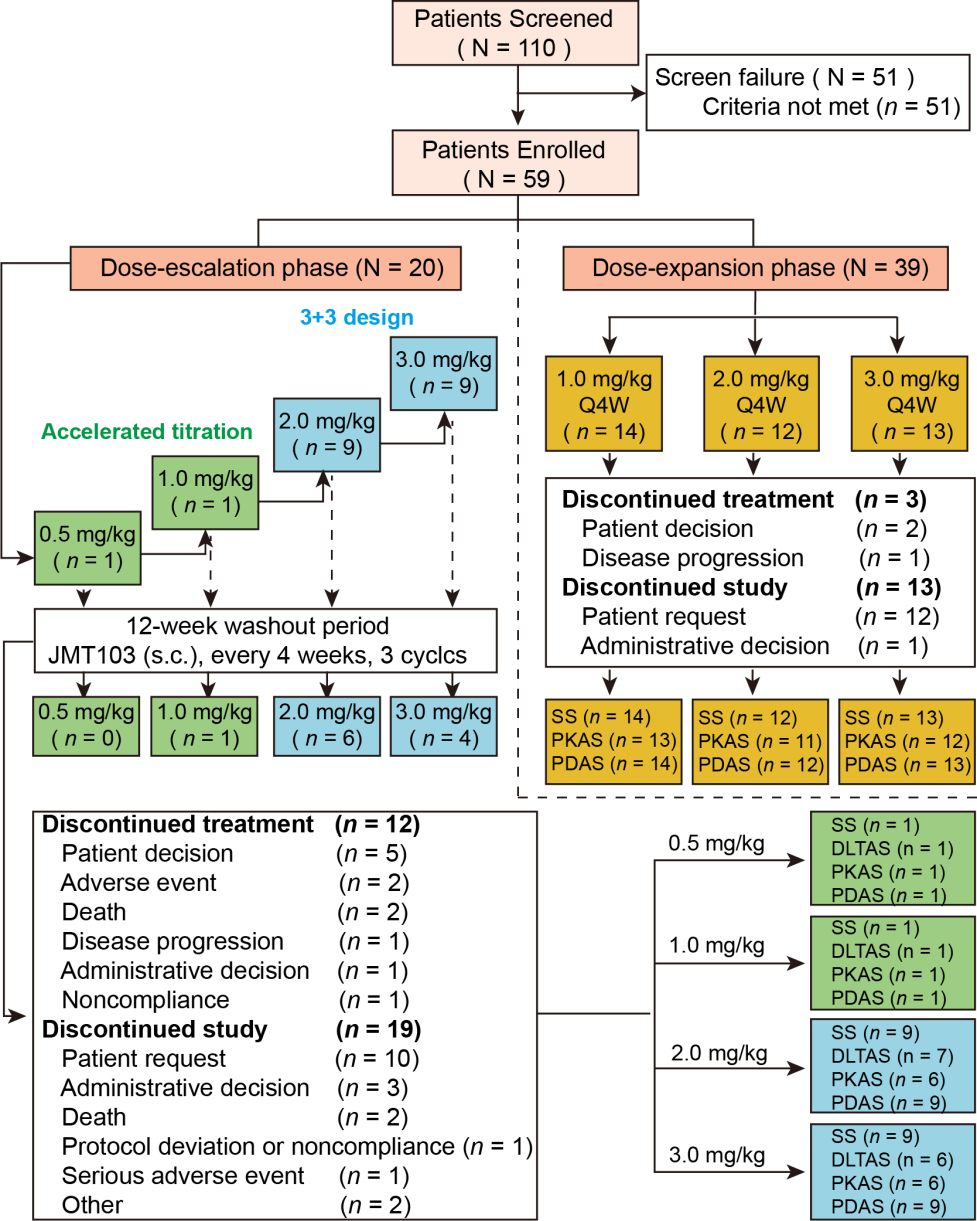
Study flow and patient disposition. SAS, safety analysis set; PKAS, PK analysis set; PDAS, PD analysis set; DLTAS, DLT analysis set; PD, pharmacodynamic; PK, pharmacokinetic; s.c., subcutaneous; Q4W, every 4 weeks.

A total of 110 Chinese patients with advanced solid tumors with bone metastases were screened between April, 2018 and September, 2020 at two sites. Of these patients, 59 were enrolled and received at least one dose of study drug: 20 in the dose-escalation phase and 39 in dose-expansion phase (**Figure 1**). The median age was 57.0 years (range, 29.0–74.0 years). In the overall population, the most frequent primary cancers were breast cancer (n = 37, 62.7%), lung cancer (n = 6, 10.2%), and gastric cancer (n = 5, 8.5%). Of all patients with bone metastasis, 38 (64.4%) had osteolytic bone metastases, 6 patients (10.2%) had osteoblastic bone metastases, and 15 (25.4%) had mixed types of bone metastasis. The demographic and baseline characteristics are summarized in **Table 1**.

**Table 1:** Demographics and baseline characteristics of 59 Chinese patients with bone metastases from solid tumors.

Characteristic	Dose-escalation phase			Dose-expansion phase				Total (*n* = 59)
	0.5 mg/kg*(n* = 1)	1.0 mg/kg(*n* = 1)	2.0 mg/kg(*n* = 9)	3.0 mg/kg(*n* = 9)	1.0 mg/kg(*n* = 14)	2.0 mg/kg(*n* = 12)	3.0 mg/kg(*n* = 13)	
Age, median (range) years	54	74	50 (29, 72)	63 (41, 73)	61 (36, 69)	56 (33, 72)	60 (29, 72)	57.0 (29, 74)
Sex (male), n (%)	1 (100.0)	1 (100.0)	3 (33.3)	3 (33.3)	2 (14.3)	2 (16.7)	3 (23.1)	15 (25.4)
Weight, median (range) kg/m^2^	63.8	77.5	47.7 (43.7, 59.5)	58.1 (38.5, 73.0)	63.4 (47.5, 94.0)	61.0 (45.5, 102.0)	66.0 (49.5, 82.3)	60.0 (38.5, 102.0)
ECOG performance status, n (%)								
0	0	0	1 (11.1)	1(11.1)	10 (71.4)	9 (75.0)	8 (61.5)	29 (49.2)
1	1 (100.0)	1 (100.0)	8 (88.9)	8 (88.9)	4 (28.6)	3 (25.0)	5 (38.5)	30 (50.8)
Original tumor, n (%)								
Breast	0	0	3 (33.3)	3 (33.3)	11 (78.6)	11 (91.7)	9 (69.2)	37 (62.7)
Lung	0	0	0	3 (33.3)	1 (7.1)	0	2 (15.4)	6 (10.2)
Gastric	0	0	4 (44.4)	1 (11.1)	0	0	0	5 (8.5)
Prostate	0	0	1 (11.1)	0	0	0	0	1 (1.7)
Rectal	1 (100.0)	0	1 (11.0)	0	0	1 (8.3)	0	3 (5.1)
Colorectal	0	1 (100.0)	0	1 (11.1)	0	0	0	2 (3.4)
Others	0	0	0	1 (11.1)^*^	2 (14.3)^§^	0	2 (15.4)^¥^	5 (8.5)
Bone metastasis type, n (%)								
Osteolytic	0	1 (100.0)	3 (33.3)	5 (55.6)	10 (71.4)	10 (83.3)	9 (69.2)	38 (64.4)
Osteoblastic	0	0	3 (33.3)	1 (11.1)	1 (7.1)	1 (8.3)	0	6 (10.2)
Mixed	1 (100)	0	3 (33.3)	3 (33.3)	3 (21.4)	1 (8.3)	4 (30.8)	15 (25.4)
Prior cancer treatment, n (%)								
Surgery	1 (100.0)	0	0	1 (11.1)	1 (7.1)	2 (16.7)	2 (15.4)	7 (11.9)
Radiotherapy^†^	1 (100.0)	0	6 (66.7)	6 (66.7)	8 (57.1)	8 (66.7)	6 (46.2)	35 (59.3)
Chemotherapy	1 (100.0)	1 (100.0)	9 (100.0)	9 (100.0)	12 (85.7)	10 (83.3)	13 (100.0)	55 (93.2)
Immunotherapy	0	0	1 (11.1)	1 (11.1)	0	1 (8.3)	0	3 (5.1)
Targeted therapy	1 (100.0)	1 (100.0)	2 (22.2)	1 (11.1)	1 (7.1)	1 (8.3)	1 (7.7)	8 (13.6)
Hormonal therapy	0	0	2 (22.2)	3 (33.3)	10 (71.4)	11 (91.7)	9 (69.2)	35 (59.3)
Other palliative therapy	0	0	1 (11.1)	0	2 (14.3)	1 (8.3)	1 (7.7)	5 (8.5)
Concomitant cancer treatment, n (%)	1 (100.0)	1 (100.0)	6 (66.7)	5 (55.6)	14 (100.0)	12 (100.0)	11 (84.6)	50 (84.7)
Hormonal therapy	0	0	3 (33.3)	4 (44.4)	9 (64.3)	11 (91.7)	9 (69.2)	36 (61.0)
Chemotherapy	0	0	4 (44.4)	3 (33.3)	12 (85.7)	9 (75.0)	6 (46.2)	34 (57.6)
Targeted therapy	1 (100.0)	1 (100.0)	0	4 (44.4)	5 (35.7)	2 (16.7)	2 (15.4)	15 (25.4)
Immunotherapy	0	0	0	1 (11.1)	0	0	0	1 (1.7)

ECOG, Eastern Cooperative Oncology Group.

^*^ Includes one patient with liver cancer;

^§^ Includes one patient with cholangiocarcinoma and one patient with nasopharynx cancer;

^¥^ Includes one patient with hidradenocarcinoma and one patient with esophageal cancer;^†^ Radiotherapy was not for bone metastasis lesions.

All patients were included in the SAS and PDAS (**Figure 1**). Thirteen patients were included in the DLTAS after excluding five patients who did not complete the 4-week DLT observation period. Eight patients were excluded from the PKAS due to inadequate data on serum JMT103 concentration and unavailability of PK parameters: six in the dose-escalation phase and two in the dose-expansion phase. Moreover, 15 patients discontinued treatment with the most common primary reason being patient decision (n = 7); 32 patients discontinued from the study with the most common primary reason being patient request (n = 22). The overall median duration of JMT103 exposure was 84 days (range 28–169 days).

During the study, 50 patients (84.7%) received concomitant cancer treatments: 36 patients (61.0%) received hormonal therapy with the most common used being fulvestrant, exemestane, anastrozole, letrozole, and tamoxifen; 34 patients (57.6%) received chemotherapy with the most common used being anthracycline, cyclophosphamide, paclitaxel, docetaxel, capecitabine, and fluorouracil; 15 patients (25.4%) received targeted therapy, mainly including apatinib and trastuzumab; and only one patient received immunotherapy (camrelizumab).

### Dose determination

The dose selection was in accordance with the results of repeated-dose toxicology and pharmacology studies (unpublished data) where there were no observed adverse effect level (10 mg/kg) and a minimum biological effect level (2.5 mg/kg), which converts to human equivalent doses of 3.2 mg/kg and 0.8 mg/kg, respectively, using allometric scaling. Taken together, the initial dose was set as 0.5 mg/kg and three dose levels of 1.0, 2.0, and 3.0 mg/kg were set for dose-expansion. Across the four dose levels explored in the dose-escalation phase, one patient (<33%) dosed with 2.0 mg/kg JMT103 developed grade 3 hypocalcemia (evaluated as a DLT by the investigator) in cycle 1, which was considered possibly related to the study drug; this patient recovered after taking Caltrate and vitamin D drops. No DLT effects occurred at doses of 0.5 mg/kg, 1.0 mg/kg, or 3.0 mg/kg. Therefore, the MTD of JMT103 has not been defined at the dose range of 0.5–3.0 mg/kg. Based on results from the dose-escalation phase, dose levels of 1.0, 2.0, and 3.0 mg/kg were selected for the dose-expansion as per protocol.

### Safety

Treatment-emergent adverse events (TEAEs) of JMT103 are summarized in **Table 2**. A total of 55 (93.2%) patients experienced at least one TEAE across both phases. The most frequent TEAEs (all grades, ≥20%) were white blood cell count decreased (50.8%), hypophosphatemia (33.9%), anemia (33.9%), aspartate aminotransferase (AST) increased (30.5%), and proteinuria (22.0%). Grade 3 or higher TEAEs occurred in 16 (27.1%) patients. The most frequent grade 3 or higher TEAEs (5%) were neutrophil count decreased (10.2%), AST increased (6.8%), and hypophosphatemia (6.8%). SAEs consisting of gastric cancer, lung neoplasm malignant, ascites, pneumonia, spinal disorder, and pleural effusion occurred in five (8.5%) patients; none of them were deemed to be TRAEs. Four patients (6.8%) discontinued the treatment because of pneumonia, hypophosphatemia, spinal disorder, and spinal cord compression. Two patients (3.4%) died, attributed to gastric cancer and lung neoplasm malignant, which were regarded as disease progressions but not treatment-related.

**Table 2:** Summary of treatment-emergent adverse events of JMT103 in the SAS.

TEAE, n (%) patients	Dose-escalation phase				Dose-expansion phase			Total(*n* = 59)
	0.5 mg/kg(*n* = 1)	1.0 mg/kg(*n* = 1)	2.0 mg/kg (*n* = 9)	3.0 mg/kg(*n* = 9)	1.0 mg/kg(*n* = 14)	2.0 mg/kg(*n* = 12)	3.0 mg/kg (*n* = 13)	
Any TEAE	1 (100)	1 (100)	9 (100)	8 (88.9)	12(85.7)	12 (100)	12 (92.3)	55 (93.2)
Grade 1	0	0	1 (11.1)	1 (11.1)	2 (14.3)	6 (50.0)	3 (23.1)	13 (22.0)
Grade 2	0	0	6 (66.7)	3 (33.3)	7 (50.0)	4 (33.3)	6 (46.2)	26 (44.1)
Grade ≥3	1 (100)	1 (100)	2 (22.2)	4 (44.4)	3 (21.4)	2 (16.7)	3 (23.1)	16 (27.1)
Serious TEAEs	1 (100) ^a^	0	0	4 (44.4) ^b^	0	0	0	5 (8.5)
TEAEs occurring with ≥3% frequency in the overall population								
WBC count decreased	0	0	5 (55.6)	3 (33.3)	6 (42.9)	9 (75.0)	7 (53.8)	30 (50.8)
Hypophosphatemia	0	1 (100)	6 (66.7)	7 (77.8)	3 (21.4)	1 (8.3)	2 (15.4)	20 (33.9)
Anemia	1 (100)	1 (100)	7 (77.8)	3 (33.3)	1 (7.1)	4 (33.3)	3 (23.1)	20 (33.9)
AST increased	1 (100)	1 (100)	3 (33.3)	2 (22.2)	4 (28.6)	3 (25.0)	4 (30.8)	18 (30.5)
Neutrophil count decreased	0	0	2 (22.2)	1 (11.1)	5 (35.7)	4 (33.3)	5 (38.5)	17 (28.8)
ALT increased	1 (100)	0	3 (33.3)	1 (11.1)	5 (35.7)	2 (16.7)	4 (30.8)	16 (27.1)
Hypocalcemia	1 (100)	1 (100)	6 (66.7)	3 (33.3)	1 (7.1)	1 (8.3)	1 (7.7)	14 (23.7)
Blood ALP increased	1 (100)	1 (100)	3 (33.3)	3 (33.3)	1 (7.1)	0	2 (15.4)	11 (18.6)
Platelet count decreased	0	0	5 (55.6)	1 (11.1)	2 (14.3)	1 (8.3)	2 (15.4)	11 (18.6)
Blood bilirubin increased	0	0	3 (33.3)	1 (11.1)	1 (7.1)	4 (33.3)	1 (7.7)	10 (16.9)
Bilirubin conjugated increased	0	0	2 (22.2)	1 (11.1)	1 (7.1)	3 (25.0)	1 (7.7)	8 (13.6)
Hemoglobin decreased	0	0	0	2 (22.2)	2 (14.3)	1 (8.3)	3 (23.1)	8 (13.6)
Blood iron decreased	0	1 (100)	3 (33.3)	1 (11.1)	1 (7.1)	1 (8.3)	0	7 (11.9)
Amylase increased	1 (100)	1 (100)	3 (33.3)	0	0	0	1 (7.7)	6 (10.2)
Protein urine present	0	0	0	3 (33.3)	1 (7.1)	0	2 (15.4)	6 (10.2)
RBC count decreased	0	0	1 (11.1)	2 (22.2)	0	1 (8.3)	2 (15.4)	6 (10.2)
Hyperglycemia	1 (100)	1 (100)	2 (22.2)	1 (11.1)	0	1 (8.3)	1 (7.7)	7 (11.9)
Hypermagnesemia	0	0	4 (44.4)	1 (11.1)	2 (14.3)	0	0	7 (11.9)
Constipation	0	0	2 (22.2)	1 (11.1)	0	3 (25.0)	1 (7.7)	7 (11.9)
Sinus bradycardia	0	0	2 (22.2)	0	2 (14.3)	2 (16.7)	0	6 (10.2)
Back pain	1 (100)	0	2 (22.2)	0	2 (14.3)	0	1 (7.7)	6 (10.2)
Proteinuria	0	1 (100)	4 (44.4)	4 (44.4)	3 (21.4)	0	1 (7.7)	13 (22.0)
TEAEs leading to treatment discontinuation	1 (100) ^a^	0	1 (11.1) ^c^	1 (11.1) ^d^	0	1 (8.3) ^e^	0	4 (6.8)
TEAEs leading to study withdrawal	1 (100) ^a^	0	1 (11.1) ^c^	2 (22.2) ^f^	0	0	0	4 (6.8)
TEAEs leading to death	0	0	0	2 (22.2) ^f^	0	0	0	2 (3.4)

SAS, safety analysis set; TEAE, treatment-emergent adverse event; WBC, white blood cell; AST, aspartate aminotransferase; ALT, alanine aminotransferase; ALP, alkaline phosphatase; RBC, red blood cell.

a Includes one patient with grade 4 pneumonia;

b Includes one patient with grade 3 ascites, one patient with grade 2 spinal disorder, one patient with grade 3 pleural effusion and grade 5 gastric cancer, and one patient with grade 5 lung neoplasm malignant;

c Includes one patient with grade 3 hypophosphatemia;

d Includes one patient with grade 2 spinal disorder;

e Includes one patient with grade 3 spinal cord compression;

f Includes one patient with grade 5 gastric cancer and one patient with grade 5 lung neoplasm malignant.

Of all the TEAEs, 29 of 59 patients (49.2%) had TEAEs that were evaluated as treatment-related AEs (TRAEs). The summary of TRAEs is listed in **Table 3**. TRAEs that occurred in more than 10% of all patients were hypophosphatemia (30.5%), hypocalcemia (23.7%), and hypermagnesemia (10.2%). Four patients (6.8%) had grade 3 or higher TRAEs (all were grade 3): hypophosphatemia (5.1%) and hypocalcemia (1.7%).

**Table 3:** Summary of treatment-related adverse events of JMT103 in the SAS.

TRAE, n (%) patients	Dose-escalation phase				Dose-expansion phase			Total(*n* = 59)
	0.5 mg/kg(*n* = 1)	1.0 mg/kg(*n* = 1)	2.0 mg/kg(*n* = 9)	3.0 mg/kg(*n* = 9)	1.0 mg/kg(*n* = 14)	2.0 mg/kg(*n* = 12)	3.0 mg/kg(*n* = 13)	
Any TRAE	1 (100)	1 (100)	9 (100)	7 77.8)	6 (42.9)	3 (25.0)	2 (15.4)	29 (49.2)
Grade ≥3	0	1 (100.0) ^a^	2 (22.2) ^b^	0	0	0	1 (7.7) ^c^	4 (6.8)
TRAEs occurring with ≥3% frequency in the overall population								
Hypophosphatemia	0	1 (100.0)	6 (66.7)	5 (55.6)	3 (21.4)	1 (8.3)	2 (15.4)	18 (30.5)
Hypocalcemia	1 (100.0)	1 (100.0)	6 (66.7)	3 (33.3)	1 (7.1)	1 (8.3)	1 (7.7)	14 (23.7)
Hypermagnesemia	0	0	4 (44.4)	0	2 (14.3)	0	0	6 (10.2)
Proteinuria	0	1 (100.0)	0	2 (22.2)	3 (21.4)	0	0	6 (10.2)
Blood iron decreased	0	1 (100.0)	2 (22.2)	0	1 (7.1)	0	0	4 (6.8)
Hypomagnesaemia	0	1 (100.0)	1 (11.1)	1 (11.1)	0	0	0	3 (5.1)
ALT increased	0	0	1 (11.1)	0	2 (14.3)	0	0	3 (5.1)
Electrocardiogram ST segment abnormal	0	0	2 (22.2)	0	1 (7.1)	0	0	3 (5.1)
Amylase increased	1 (100.0)	0	1 (11.1)	0	0	0	0	2 (3.4)
AST increased	0	0	0	0	2 (14.3)	0	0	2 (3.4)
Blood ALP increased	0	0	0	0	1 (7.1)	0	1 (7.7)	2 (3.4)
Blood parathyroid hormone increased	1 (100.0)	1 (100.0)	0	0	0	0	0	2 (3.4)
Pain in extremity	0	0	0	1 (11.1)	1 (7.1)	0	0	2 (3.4)

SAS, safety analysis set; TRAE, treatment-emergent adverse event; ALT, alanine aminotransferase; AST, aspartate aminotransferase; ALP, alkaline phosphatase.

a Includes one patient with grade 3 hypophosphatemia;

b Includes one patient with grade 3 hypophosphatemia and one patient with grade 3 hypocalcemia;

c Includes one patient with grade 3 hypophosphatemia.

There were no clinically significant findings in coagulation function, peripheral lymphoid classification, vital signs, physical examination, ECG, or oral examination. Clinical abnormalities in hematology and blood biochemistry were irregular, and were uncorrelated with JMT103 administration. Eight patients were excluded from the immunogenicity analysis due to the lack of post-dose ADA results, and only one patient (2.0%) in the 2.0 mg/kg group in the dose-expansion phase tested positive for anti-JMT103 antibodies at day 1 pre-dose and day 57 pre-dose. All the other patients tested negative for anti-JMT103 antibodies.

### Pharmacokinetics

**Figure 2:**
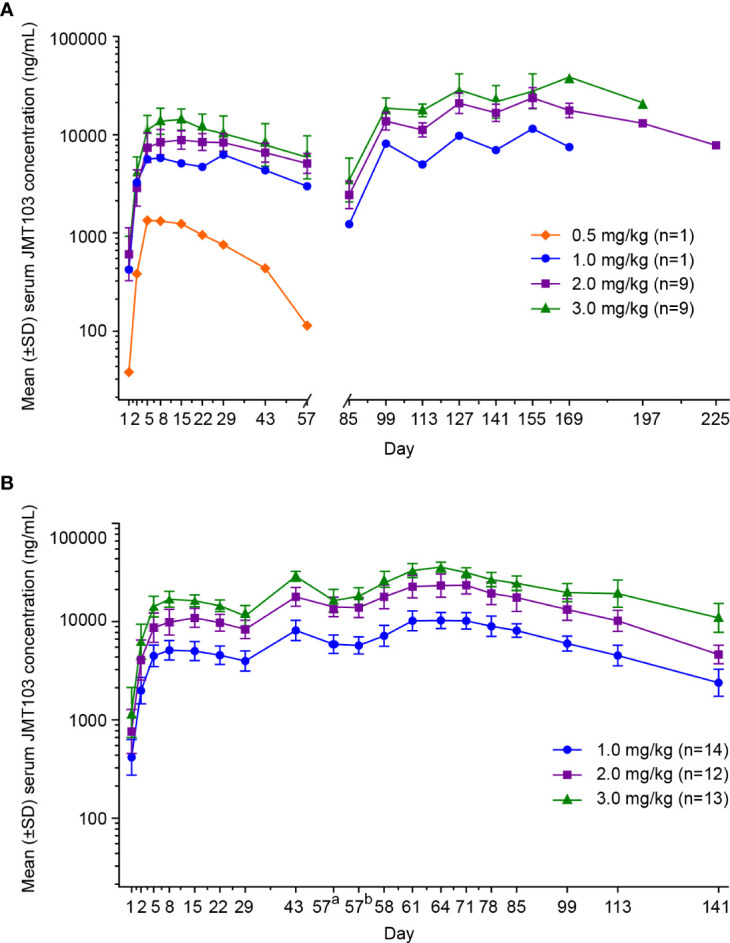
Mean concentration-time profiles following subcutaneous administration of JMT103 in patients with bone metastasis from solid tumors. **(A)** Serum concentration-time profile of JMT103 after single-dose administration; **(B)** serum concentration-time profile of JMT103 after multiple-dose administration. SD, standard deviation. ^a^ means pre-dose;^b^ means post-dose.

In the dose-escalation phase, the mean serum concentration-time profiles of JMT103 after a single dose are shown in **Figure 2A**. The detailed PK parameters are summarized in **Table 4**. JMT103 was absorbed slowly after a single dose, with a median time to maximum serum concentration (T_max_) ranging from 3.98–27.80 days, whereas absorption was slower for the intermediate dose (1.0 mg/mg with the T_max_ of 27.80 days). Thereafter, JMT103 concentrations decreased slowly, with the mean half-life (t_1/2_) ranging from 13.30–28.90 days. Exposure (maximum serum concentration (C_max_), area under the serum concentration-time curve [AUC]_0-28day_, AUC_0-t_, and AUC_0-inf_) of JMT103 showed a dose-proportional increase across the doses from 0.5 to 3.0 mg/kg after the first dose (**Table 5**).

**Table 4:** Summary of plasma pharmacokinetic parameters of JMT103 after single and multiple doses in the PKAS.

PK parameters	Dose cohorts			
	0.5 mg/kg	1.0 mg/kg	2.0 mg/kg	3.0 mg/kg
After a single dose in dose-escalation phase				
C_max_ (ng/mL)	1340	6250	10100 (2010)	14800 (5200)
T_max_ (day)	3.98	27.80	10.48 (3.98-27.97)	6.96 (6.92-20.99)
t_1/2_ (day)	13.30	23.50	28.90 (6.63)	22.30 (6.02)
AUC_0-28day_ (μg*h/mL)	706	3450	5580 (1270)	7870 (3040)
AUC_0-t_ (μg*h/mL)	1050	7770	12300 (2570)	14500 (7920)
AUC_0-inf_ (μg*h/mL)	1100	8770	13700 (2500)	14500 (7110)
λ_z_ (1/day)	0.05	0.03	0.03 (0.01)	0.03 (0.01)
CL/F (L/day)	0.70	0.21	0.19 (0.02)	0.36 (0.21)
V_z_/F (L)	13.40	7.20	7.88 (1.71)	10.30 (2.74)
After a single dose in dose-expansion phase				
C_max_ (ng/mL)	–	5290 (1220)	11200 (2940)	17500 (3080)
T_max_ (day)	–	6.96 (3.93-21.96)	13.88 (6.86-13.98)	6.93 (3.94-28.89)
AUC_0-28day_ (μg*h/mL)	–	2980 (494)	6630 (1790)	9520 (1410)
AUC_0-t_ (μg*h/mL)	–	3000 (742)	6300 (1580)	10500 (2600)
After multiple doses in dose-expansion phase				
C_max_ (μg/mL)	–	11.50 (1.86)	25.30 (5.71)	35.10 (5.13)
C_min_ (μg/mL)	–	6.27 (0.84)	14.00 (2.76)	19.10 (1.05)
C_avg_ (μg/mL)	–	9.88 (1.58)	21.70 (5.31)	28.90 (4.12)
T_max_ (day)	–	6.88 (3.89-21.90)	6.93 (3.87-13.92)	6.95 (1.99-13.93)
t_1/2_ (day)	–	32.00 (8.22)	32.30 (5.25)	33.40 (9.09)
AUC_0-t_ (μg*h/mL)	–	13000 (2380)	27200 (5810)	31300 (11100)
AUC_τ_ (μg*h/mL)	–	6640 (1060)	14600 (3570)	19400 (2770)
λ_z_ (1/day)	–	0.023 (0.005)	0.022 (0.004)	0.022 (0.006)
CL/F (L/day)	–	0.25 (0.052)	0.24 (0.080)	0.26 (0.062)
V_z_/F (L)	–	11.40 (3.10)	11.20 (4.03)	12.60 (4.87)
Ra_(AUC0-t)_	–	2.11 (0.49)	2.22 (0.24)	2.08 (0.37)
Ra_(Cmax)_	–	2.11 (0.47)	2.27 (0.34)	2.03 (0.37)

Data are expressed as mean (SD) or as median (range) for T_max_. One patient in the 2.0 mg/kg cohort and two patients in the 3.0 mg/kg cohort were excluded from the calculation of AUC_0-inf_, λz, t1/2, CL/F, and V_z_/F, because their %AUCex were more than 20%. %AUC_ex_, [(AUC_0-inf_ - AUC_0-t_)/AUC_0-in_f] *100%.

PKAS, pharmacokinetic analysis set; PK, pharmacokinetic; C_max_, maximum serum concentration; T_max_, time to peak serum concentration; t_1/2_, terminal elimination half-life; AUC, area under the serum concentration-time curve; AUC_0-28day_, AUC from time zero to 28 days; AUC_0-t_, AUC from time zero (pre-dose) to last time of quantifiable concentration; AUC_0-inf_, AUC from time zero to infinite time; AUC_τ_, AUC in a dose interval; λ_z_, elimination rate constant; CL/F, apparent clearance; V_z_/F, apparent volume of distribution; C_min_, minimum serum concentration; C_avg_, average plasma concentration; Ra_(AUC0-t)_, accumulation ratio, which was calculated as the ratio of AUC_0-t_ after multiple doses to AUC_0-t_ after a single dose; Ra_(Cmax)_, accumulation ratio, which was calculated as the ratio of C_max_ after multiple doses to C_max_ after a single dose.

**Table 5:** Dose proportionality assessment of JMT103.

PK parameters	Dose range (mg/kg)	Sample size	Estimated values (β1)	90% CI
After a single-dose in the dose-escalation phase				
C_max_	0.5-3.0	14	1.17	0.86, 1.47
AUC_0-28day_		14	1.16	0.81, 1.50
AUC_0-t_		14	1.15	0.65, 1.65
AUC_0-inf_		11	1.18	0.61, 1.74
After a single-dose in the dose-expansion phase				
C_max_	1.0-3.0	37	1.10	0.96, 1.25
AUC_0-28day_		24	1.06	0.93, 1.19
AUC_0-t_		37	1.15	0.99, 1.30
After multiple doses in the dose-expansion phase				
C_max_	1.0-3.0	25	1.03	0.90, 1.17
C_avg_		25	1.00	0.86, 1.14
C_min_		25	1.04	0.93, 1.16
AUC_τ_		25	1.00	0.86, 1.15
AUC_0-t_		25	0.81	0.60, 1.02

Dose proportionality was confirmed when the 90% CI of the β1 value contained the value 1.00.

CI, confidence interval; PK, pharmacokinetic; C_max_, maximum serum concentration; AUC, area under the serum concentration-time curve; AUC_0-28day_, AUC from time zero to 28 days; AUC_0-t_, from time zero (pre-dose) to last time of quantifiable concentration; AUC_0-inf_, AUC from time zero to infinite time; C_avg_, average plasma concentration; C_min_, minimum serum concentration; AUC_τ_, AUC in a dose interval.

In the dose-expansion phase, the mean serum concentration-time profiles are shown in **Figure 2B**. The PK parameters of JMT103 after a single dose and multiple doses are summarized in **Table 4**. After a single dose, median T_max_ in the 2.0 and 3.0 mg/kg cohorts were comparable to those values observed in the dose-escalation phase. C_max_ and AUC_0-t_ increased in proportion to the doses ranging from 1.0 to 3.0 mg/kg, which were consistent with the results in the dose-escalation phase (**Table 5**). After multiple doses, the median T_max_ ranged from 6.88–6.95 days and the mean t_1/2_ ranged from 32.0–33.4 days. Median T_max_ and mean t_1/2_ were not dose dependent. C_max_ and AUC_τ_ showed a dose-proportional increase across the doses ranging from 1.0 to 3.0 mg/kg. Limited accumulation of JMT103 was observed, with accumulation rates of 2.03–2.27 for the different doses when analyzing the accumulation ratio (Ra)_(AUC0-t)_ and Ra_(Cmax)_.

### Pharmacodynamics

Reductions in uNTx/Cr, sCTX-I, TRAP5b, bALP, and albumin-adjusted serum calcium were observed at the first visit after the first dose, and the magnitude of reductions in uNTx/Cr, sCTX-I, and TRAP5b were substantial and maintained throughout the study (**Table 6**).

**Table 6:** Median percentage changes from baseline in bone turnover biomarkers in the PDAS.

Bone turnover biomarkers	Dose-escalation phase			Dose-expansion phase		
	1.0 mg/kg	2.0 mg/kg	3.0 mg/kg	1.0 mg/kg	2.0 mg/kg	3.0 mg/kg
uNTx/Cr (nM BCE/mM) ^a^−median (IQR)						
Baseline	144.0	153.0 (90.5, 262.7)	28.6 (3.3, 67.0)	49.6 (35.6, 108.4)	24.2 (17.6, 32.1)	42.4 (27.3, 87.2)
Day 2	-74.1	-70.0 (-77.7, -53.3)	-61.6 (-64.7, -33.3)	-41.7 (-71.8, -19.3)	-32.9 (-56.7, -10.3)	-38.6 (-72.7, 24.4)
Day 15	-81.3	-87.6 (-88.5, -81.2)	-47.4 (-67.2, -17.9)	-92.5 (-95.9, -77.5)	-35.2 (-69.9, -18.1)	-75.0 (-98.5, -60.3)
Day 85	-95.1	-95.3 (-99.1, -85.5)	-7.77 (-52.5, 405.4)	-78.6 (-85.3, -71.8)	-48.2 (-69.4, 14.7)	-97.9 (-98.5, -30.8)
Day 141	-92.8	-94.7 (-98.5, -91.6)	159.6 (-10.3, 329.0)	-74.8 (-80.7, -31.9)	-61.4 (-93.4, -16.4)	-92.2 (-95.6, -76.5)
sCTx-I (ng/mL)−median (IQR)						
Baseline	0.5	1.0 (0.5, 1.5)	0.4 (0.3, 0.6)	0.4 (0.3, 0.5)	0.4 (0.2, 0.6)	0.4 (0.2, 0.5)
Day 2	-68.5	-54.0 (-72.7, -50.6)	-52.2 (-58.5, -13.0)	-60.0 (-65.6, -38.5)	-48.3 (-58.8, -20.7)	-58.8 (-66.9, -44.7)
Day 15	-92.6	-88.0 (-92.6, -84.8)	-30.8 (-82.6, -21.7)	-78.1 (-85.7, -58.6)	-38.9 (-68.4, -27.4)	-66.7 (-85.9, -36.3)
Day 85	-83.3	-83.2 (-88.6, -77.2)	-43.0 (-67.3, 46.6)	-70.0 (-76.5, -39.1)	-60.1 (-72.3, -35.7)	-81.5 (-87.2, -66.8)
Day 141	-85.2	-83.8 (-86.1, -72.7)	43.1 (-60.9, 147.0)	-28.0 (-53.3, 11.1)	-54.9 (-82.6, 0.0)	-72.6 (-78.2, -48.4)
TRAP5b (U/L)−median (IQR)						
Baseline	6.0	5.1 (4.5, 8.5)	4.1 (3.2, 5.1)	3.2 (2.8, 5.0)	3.9 (2.8, 6.0)	5.3 (3.6, 6.5)
Day 2	-11.3	-2.9 (-4.0, 1.2)	-10.8 (-13.7, -6.8)	-9.6 (-14.3, -6.4)	0.0 (-5.9, 3.6)	-12.4 (-20.3, -3.5)
Day 15	-66.6	-54.1(-59.8, -46.4)	-37.6 (-48.6, -32.8)	-23.5 (-48.7, -18.9)	-27.5 (-49.4, -9.8)	-45.7 (-48.0, -42.4)
Day 85	-37.3	-51.3 (-58.5, -32.1)	-28.9 (-44.1, -1.0)	-26.7 (-44.8, 0.3)	-23.0 (-52.2, 4.3)	-43.3 (-52.5, -33.7)
Day 141	-79.8	-53.0 (-73.9, -43.8)	-19.1 (-56.8, 18.5)	-23.8 (-36.9, 1.1)	-24.9 (-43.8, 0.4)	-14.5 (-34.6, 13.4)
bALP (μg/L)−median (IQR)						
Baseline	33.3	48.3 (30.6, 168.0)	10.4 (10.0, 11.0)	14.2 (10.6, 22.8)	17.8 (9.0, 26.8)	19.9 (12.3, 21.2)
Day 2	-23.0	-6.6 (-12.5, -4.8)	-6.7 (-10.0, 8.8)	-0.7 (-5.5, 6.7)	-1.0 (-5.6, 1.8)	-3.2 (-10.3, 5.5)
Day 15	-23.4	-30.3 (-44.7, -9.4)	17.4 (-14.8, 20.1)	5.2 (-0.3, 11.9)	-6.2 (-10.3, 3.5)	0.7 (-14.7, 13.5)
Day 85	-57.8	-63.4 (-71.5, -50.1)	11.4 (-25.4, 94.7)	-13.5 (-30.6, 1.1)	-25.4 (-34.7, 9.4)	-52.8 (-56.9, -35.9)
Day 141	-15.7	-76.7 (-78.6, -42.4)	49.3 (-21.2, 119.9)	-27.3 (-49.0, -16.0)	-31.4 (-40.5, -23.8)	-45.9 (-63.2, -13.5)
iPTH (pmol/L)−median (IQR)						
Baseline	4.2	3.2 (2.9, 5.9)	3.5 (1.5, 4.3)	3.3 (2.6, 4.0)	4.4 (2.3, 5.7)	4.3 (3.1, 6.1)
Day 2	32.0	54.3 (49.7, 90.7)	24.1 (-9.6, 27.8)	37.0 (15.2, 73.0)	1.05 (-10.5, 59.4)	28.8 (-1.9, 87.5)
Day 15	159.0	207.1 (149.4, 286.4)	101.0(37.7, 176.8)	91.1 (20.7, 126.3)	23.4 (3.8, 53.1)	59.4 (17.3, 97.7)
Day 85	106.0	109.7 (27.3, 217.4)	24.6 (-20.2, 59.6)	71.0 (19.8, 100.0)	42.7 (24.2, 56.7)	30.9 (23.6, 36.9)
Day 141	93.3	102.0 (17.1, 123.4)	-31.0 (-31.8, -30.2)	63.3 (34.1, 93.4)	65.6 (20.5, 129.6)	10.0 (-21.0, 14.3)
adjCa (mg/dL)−median (IQR)						
Baseline	9.1	9.3 (8.9, 9.5)	9.2 (8.9, 9.7)	9.5 (9.3, 9.7)	9.3 (9.3, 9.6)	9.4 (9.3, 9.7)
Day 2	-2.6	-6.8 (-12.6, -4.6)	-2.5 (-7.1, -0.4)	-2.9 (-5.0, -0.9)	-1.9 (-4.8, 0.8)	-4.5 (-5.7, -2.7)
Day 15	-8.8	-10.1 (-16.5, -5.1)	-5.5 (-9.0, 5.0)	-2.1 (-7.3, 0.4)	0.9 (-4.3, 3.8)	-2.8 (-4.9, 0.0)
Day 85	0.0	-4.2 (-8.8, -1.8)	-3.8 (-6.3, 3.8)	-1.7 (-3.7, 3.0)	-2.8 (-5.1, 0.8)	-3.5 (-7.0, -0.4)
Day 141	-10.3	-0.5 (-3.4, -0.4)	-1.3 (-7.5, 4.9)	-1.7 (-3.9, 1.3)	-1.9 (-3.8, 1.8)	0.6 (-0.4, 5.1)

PDAS, pharmacodynamic analysis set; uNTx, urinary N-telopeptide; Cr, urinary creatinine; BCE, bone collagen equivalents; sCTx-I, serum C-telopeptide I; TRAP5b, tartrate-resistant acid phosphatase 5b; bALP, bone alkaline phosphatase; iPTH, intact parathyroid hormone; adjCa, serum albumin-adjusted serum calcium; IQR, interquartile range, quartile that refers to the first quartile and the third quartile.

a uNTx/Cr is reported in nmol of bone collagen equivalents/mmol creatinine.

**Figure 3:**
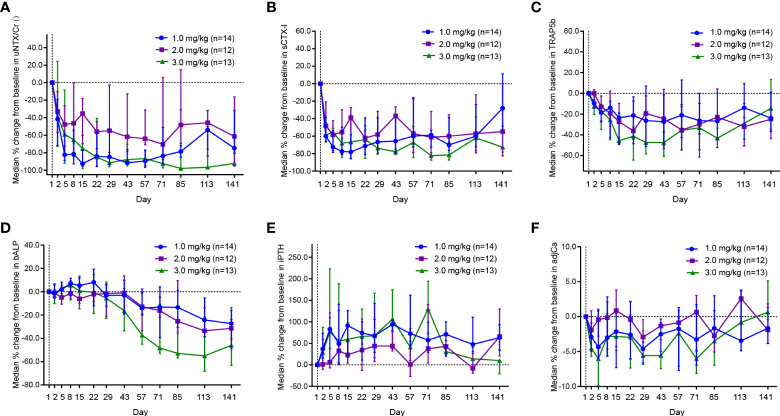
Effect of JMT103 on the concentration of **(A)** uNTx/Cr; **(B)** sCTx-I; **(C)** TRAP5b; **(D)** bALP; **(E)** iPTH; **(F)** adjCa. uNTx, urinary N-telopeptide; Cr, urinary creatinine; sCTx-I, serum C-telopept. TRAP5b, tartrate-resistant acid phosphatase 5b; bALP, bone alkaline phosphatase; iPTH, intact parathyroid hormone; adjCa, serum albumin-adjusted serum calcium.

The changes in bone turnover biomarkers in the dose-expansion phase are shown in **Figure 3**. The median uNTx/Cr concentrations for the 1.0 and 3.0 mg/kg dose cohorts decreased over 60.0% within 8 days and were maintained up to day 141 (**Figure 3A**). At day 141, the median uNTx/Cr concentration percentage changes from baseline were –74.8% (interquartile range [IQR], –80.7% to –31.9%) in the 1.0 mg/kg cohort, –61.4% (IQR, –93.4% to –16.4%) in the 2.0 mg/kg cohort, and –92.2% (IQR, –95.6% to –76.5%) in the 3.0 mg/kg cohort. The maximum uNTx/Cr concentration percentage change from baseline was 97.9% (IQR, –98.5% to –30.8%), occurring in the 3.0 mg/kg cohort at day 85.

At day 2, the median sCTX-I concentration percentage changes from baseline were –60.0% (IQR, –65.6% to –38.5%), –48.3% (IQR, –58.8% to –20.7%), and –58.8% (IQR, –66.9% to –44.7%) in the 1.0 mg/kg, 2.0 mg/kg, and 3.0 mg/kg cohorts, respectively (**Figure 3B**). In the 3.0 mg/kg cohort, this magnitude of decrease (≥60.0%) was maintained throughout the study. The maximum median sCTX-I concentration percentage changes from baseline were –78.1% (IQR, –85.7% to –58.6%) in the 1.0 mg/kg cohort at day 15, –62.1% (IQR, –67.2% to –53.5%) in the 2.0 mg/kg cohort at day 22, and –81.5% (IQR, –87.2% to –66.8%) in 3.0 mg/kg cohort at day 85.

The median TRAP5b concentration decreased rapidly during the first cycle (**Figure 3C**). At day 15, the TRAP5b concentration in the 3.0 mg/kg cohort reached the nadir with a median 45.7% decrease. The decrease was maintained at around 20% during 3 cycles. At day 141, the median TRAP5b concentration percentage changes from baseline were –23.8% (IQR, –36.9% to 1.1%) in the 1.0 mg/kg cohort, –24.9% (IQR, –43.8% to 0.4%) in the 2.0 mg/kg cohort, and –14.5% (IQR, –34.6% to 13.4%) in the 3.0 mg/kg cohort.

Although the median bALP concentration in the 1.0 mg/kg and 3.0 mg/kg cohorts increased slightly at first, the trends began to reverse on days 8 and 22 (**Figure 3D**). The decreasing trends continued until the end of the study (up to day 141) and reached the maximum reduction on day 113. On the contrary, the median iPTH concentration in the 1.0 and 3.0 mg/kg cohorts increased greatly (over 70%) from day 1 to day 5 (**Figure 3E**). In the 1.0 mg/kg cohort, this large degree of promotion remained over the course of the study. The maximum median increase in iPTH concentration was 130.3% (IQR, 73.2% to 194.5%), occurring in the 3.0 mg/kg cohort at day 71. During the study, the median albumin-adjusted serum calcium concentrations decreased slightly and remained within 6.0% from baseline. Although the maximum albumin-adjusted serum calcium concentration percentage change from baseline was observed in the 3.0 mg/kg cohort on day 71, the decrease trended toward baseline thereafter (**Figure 3F**).

## Discussion

This research was on the safety, tolerability, and PK/PD of JMT103 in Chinese patients with bone metastases from solid tumors. The safety assessments indicated that JMT103 was well tolerated in all dose cohorts after single and multiple doses, and no significant safety signals were identified. Only one (14.3%) patient in the 2.0 mg/kg dose escalation group reported grade 3 hypocalcemia, which was evaluated as a DLT by the investigator. After giving Caltrate and vitamin D drops, remission was achieved. Twenty-nine (49.2%) patients reported at least one TRAE throughout the study, most of which were mild or moderate in severity. Incidences of hypophosphatemia and hypocalcemia, two of the most common TRAEs, were 30.5% and 23.4%, respectively. This was consistent with the AE profile of denosumab in three trials, in which the incidence of hypophosphatemia and hypocalcemia were 32% and 18%, respectively, in a total of 2841 patients with bone metastasis from solid tumors (21–23). Moreover, severe hypocalcemia (corrected serum calcium <7 mg/dL) occurred in one patient (1.7%), which was half that of patients treated with denosumab (3.1%), as described in the full prescribing information (24). Two deaths occurred during the study, but neither was deemed to be treatment-related. In addition, the ADA positivity rate of JMT103 (2.0%) was slightly higher than that of denosumab, for which no patients tested positive for anti-denosumab antibodies, as reported in previous publications (25–28). Nonetheless, the ADA positivity rate was very low; it is also worth noting that the anti-JMT103 antibody was positive twice, on day 1 pre-dose and day 57 pre-dose, but negative at all other timepoints post-dose. We speculated that the positive ADA results were unrelated to the study-drug, and were probably false positives, or the patient may have had antibodies with a similar target before enrollment and off the record. Overall, doses up to 3.0 mg/kg JMT103 were well tolerated without definitive dose-related incidence and severity of TEAEs.

The PK parameters showed that JMT103 was absorbed slowly and sustained with a median T_max_ of 6.96–27.80 days and mean t_1/2_ of 22.30–28.90 days at a single dose range from 1.0 to 3.0 mg/kg, and a median T_max_ of 6.88–6.95 days and a mean t_1/2_ of 32.00-33.40 days at multiple doses ranging from 1.0 to 3.0 mg/kg. The PK profile after a single dose was similar to that of denosumab in Japanese women with bone metastases from breast cancer, which showed a median T_max_ of 8–10 days and mean t_1/2_ of 25–29 days at a single dose ranging from 60 to 180 mg (29). The median T_max_ of JMT103 at multiple doses was more than half that observed with denosumab, which showed a median T_max_ of 14–18 days after 180 mg (Q4W) dosing. Moreover, as with denosumab administered at weight-adjusted doses from 0.03 to 3.0 mg/kg in patients or in healthy participants (25, 27), which demonstrated an approximately linear PK profile at doses ranging from 60–180 mg (28, 29), the serum JMT103 concentration increased in an approximately dose-proportional manner in single- and multiple-dose studies. The difference may be caused by the sampling timepoint selection.

As previously stated, bone metastases are characterized by elevated bone turnover markers such as the uNTx/Cr; patients with elevated levels of uNTx/Cr are at increased risk for SREs, disease progression, and death (30–32). The suppression of bone resorption biomarkers (including uNTx/Cr, sCTX-I, and TRAP5b) and bone formation biomarker (bALP) were significant and sustained. In particular, the suppression of uNTx/Cr was rapid and dramatic, with an 80% median reduction observed at week-2 after the initial doses of 1.0 and 2.0 mg/kg (in the dose-escalation phase). Suppression of uNTx/Cr occurred at all doses of JMT103 and continued through day 141; this was consistent with that of denosumab from previous studies, which sustainedly suppressed uNTx/Cr throughout the entire dosing interval in every 4-week cohort (60, 120, or 180 mg, Q4W) (26, 33). Additionally, the duration of maximal uNTx/Cr suppression increased with denosumab dosing (30 to 180 mg, Q4W) (25). although it was not dependent on the JMT103 dosing in this study. It might be related to the lower baseline uNTx/Cr concentration in the 2.0 mg/kg dose cohort compared with that in the 1.0 and 3.0 mg/kg dose cohorts. Overall, JMT103 can suppress markers of bone turnover to a level similar to that previously reported for denosumab in patients with bone metastases from solid tumors. This indicates that JMT103 might be a promising treatment for bone loss caused by bone metastases, multiple myeloma, or osteoporosis.

Limitations of this study are the small sample size, absence of a control group, and inclusion of selected patients with different concomitant therapies and primary tumors exhibiting different types of bone metastasis lesions: osteolytic, osteoblastic, and mixed type. It is unknown whether such differences affect the safety, PK, and PD of the study medication. Most commonly, osteoblastic metastases are seen in prostate cancer; most other solid tumors, including lung, thyroid, breast, and kidney malignancies, tend to form osteolytic metastases (34). It also must be noted that both bone resorption and formation to different degrees can be seen in most cancers (2, 8). A systematic review suggested that neither tumor type nor type of concomitant therapy markedly affects denosumab PK or PD across breast, prostate, and other solid tumors with bone metastasis (35). Additionally, there were almost no individual differences in the PK profile in this study. Taken together, we believe that the effect of the bone metastasis type and concomitant cancer treatments on the safety and PK of JMT103 is minimal. Another limitation is the potential bias that all included patients were Chinese. Although racial disparities exist in the prevalence, biologic mechanism, development, SREs, and outcomes of bone metastasis (36–38), to date, no reports have shown the differences in safety, tolerability, PK, and PD of denosumab among the different ethnic populations (24). Further research is needed to confirm this issue for JMT103.

Finally, in the PD analysis, the limited number of biomarker-evaluable patients in this study precludes drawing a statistically certain conclusion, but these compelling results allow us to initiate a randomized, single-blind, placebo-controlled study to further inform the therapeutic potential of JMT103 in patients with bone metastases from solid tumors (NCT04630522). Another two studies aiming to evaluate the efficacy and safety of JMT103 in patients with giant cell tumor of bone (NCT04255576) and refractory hypercalcemia of malignancy (NCT04198480) are ongoing, and the former study has preliminary results showing that JMT103 has potential anti-tumor efficacy in giant cell tumor of bone, with a tumor response rate of 81.3% (39). Recently, some results reported that vascular endothelial growth factor (VEGF) and mTOR are involved in RANKL-induced osteoclastogenesis (40, 41). Lenvatinib (a TKI inhibitor targeting VEGF receptor) has shown promising antitumor activity in osteosarcoma and giant cell tumor of bone and desmoplastic fibroma (42, 43). Furthermore, everolimus (an mTOR inhibitor) is proved to be beneficial in decreasing bone resorption and exerting a bone protective effect in metastatic breast cancer (44). Notably, the combination of denosumab with everolimus was showed to be more effective than denosumab alone in reducing osteoclast differentiation *in vitro* studies (41). These results provided a promising therapeutic strategy in bone diseases and highlighted the future research direction of JMT103.

In conclusion, this phase 1 study shows that JMT103 has a good safety profile and potential clinical activity in patients with bone metastases from solid tumors. The PK and PD profiles of JMT103 appear to be similar to those of denosumab. JMT103 is a promising new treatment option for patients with bone metastases from solid tumors, and other diseases that cause abnormal bone metabolism, such as giant cell tumors of bone, multiple myeloma, and osteoporosis.

## Data availability statement

The raw data supporting the conclusions of this article will be made available by the authors, without undue reservation.

## Ethics statement

The studies involving human participants were reviewed and approved by ethics committee of Peking University Cancer Hospital & Institute, and ethics committee of Shanghai East Hospital. The patients/participants provided their written informed consent to participate in this study.

## Author contributions

XL, JX, JL, HL, YG, XY, ZW, TZ, and CW conceived and designed the study and collected data. XL, JX, LX, LD, WT, GS, QL, HJ, WZ, FL, and BS collected data and wrote the manuscript. All authors analyzed and interpreted the data and were involved in the development, review, and approval of the manuscript.

## Funding

The study was sponsored by Shanghai JMT-BIO technology Co., Ltd.

## Acknowledgments

We thank the patients and their families for participating in this study, and the investigators and research staff at all study sites for their contributions. We also acknowledge Baoxia Zhang, Lei Wang, and Xiaoning Yang for providing editorial and medical writing assistance.

## Conflict of interest

XY, ZW, TZ, and CW were employed by CSPC ZhongQi Pharmaceutical Technology (Shijiazhuang) Co., Ltd. JL serves as a consultant to Hutchison, Eli Lilly, and CTTQ. YG received speaker honoraria from Merck Serono, Roche, MSD, BMS, and serves on scientific advisory board for Merck Serono, MSD, Bayer, and Roche.

The remaining authors declare that the research was conducted in the absence of any commercial or financial relationships that could be construed as a potential conflict of interest.

The authors declare that this study received funding from Shanghai JMT-BIO technology Co., Ltd. The funder had the following involvement with the study: study design, collection, analysis, interpretation of data, and the decision to submit it for publication.

## Publisher’s note

All claims expressed in this article are solely those of the authors and do not necessarily represent those of their affiliated organizations, or those of the publisher, the editors and the reviewers. Any product that may be evaluated in this article, or claim that may be made by its manufacturer, is not guaranteed or endorsed by the publisher.
